# A meta-analysis of the factors influencing development rate variation in *Aedes aegypti* (Diptera: Culicidae)

**DOI:** 10.1186/1472-6785-14-3

**Published:** 2014-02-05

**Authors:** Jannelle Couret, Mark Q Benedict

**Affiliations:** 1 Department of Biology, Emory University, 1510 Clifton Road NE, Atlanta, GA 30322, USA; 2 Dipartimento di Medicina Sperimentale e Scienze Biochimiche, Sezione di Microbiologia, Edificio B, 3 Piano, Via Gambuli, 06132 Perugia, Italy

**Keywords:** Mosquito, Meta-analysis, Temperature, Development, Diet, Density, Development rate, Survival

## Abstract

**Background:**

Development rates of *Aedes aegypti* are known to vary with respect to many abiotic and biotic factors including temperature, resource availability, and intraspecific competition. The relative importance of these factors and their interactions are not well established across populations. We performed meta-analysis on a dataset of development rate estimates from 49 studies.

**Results:**

Meta-analytic results indicated that the environmental factor of temperature is sufficient to explain development rate variability in *Ae. aegypti*. While diet and density may greatly impact other developmental phenotypes, these results suggest that for development rate these factors should never be considered to the exclusion of temperature. The effect of temperature on development rate is not homogenous or constant. The sources of heterogeneity of the effect of temperature are difficult to analyze due to lack of consistent reporting of larval rearing methods.

**Conclusions:**

Temperature is the most important ecological determinant of development rate in *Ae. aegypti,* but its effect is heterogeneous. Ignoring this heterogeneity is problematic for models of vector population and vector-borne disease transmission.

## Background

The effect of temperature on growth has been studied across a wide diversity of organisms [1-6]. Like all poikilotherms, the biochemical and physiological processes of insects depend on body temperature, and ambient environmental temperature has a profound effect on the metabolic rate and growth of insects. With short generation times and high fecundity, insects are convenient model species both in the laboratory and the field, as over a century of research establishes that temperature influences the duration and rate of development [7-16]. A main feature of this body of research is the emphasis on prediction of the timing of maturation [17,18], body size [19,20], and population dynamics [21-23]. However, with the benefits of simplicity and practicality of considering only temperature for predicting developmental timing come the costs of ignoring other environmental and ecological factors of known importance such as resource availability, competition, and predation.

Particularly in insects of medical importance, such as mosquitoes that vector human pathogens, estimates of developmental characteristics and models of developmental timing are used to guide vector population control efforts [24]. In particular, controlling the population of the mosquito vector *Aedes aegypti* (Linnaeus) is critical to preventing dengue infection [25], as there is no vaccine or chemotherapeutic treatment [26]. In *Ae. aegypti,* insecticide resistance [27,28] and continued progress with transgenic strains and their release [29] underscore the need to understand developmental phenotypes. Increasingly unpredictable climate patterns [30] motivate the study of development rate in response to varied environmental conditions [31,32].

Few studies have sought to determine importance of other conditions of the developmental environment relative to temperature to explain individual variation in development rate [7,33]. Plasticity of development rate has been demonstrated in many diverse taxa. In mosquitoes, developmental traits vary in response to gradients of abiotic and biotic factors such as diet [34-41], larval rearing density [24,42,43], fungal infection [44], nutrient quality [45,46], thermoperiodism [47], and presence of predators [48]. Inclusion of the variability in development rate with respect to factors other than temperature might improve the realism of models. However, temperature is often considered the main driver of development [49], and it is unclear whether other factors are necessary to adequately explain variation in development rate. We hypothesize that development rate is significantly influenced by several environmental factors apart from temperature and that the interaction of these factors is an important predictor of development rate variation.

To test these hypotheses in diverse environmental conditions, empirical data is needed that considers development rate 1) in response to multiple factors [50], 2) over a gradient, (i.e. 2 or more levels) of each environmental factor [17], and 3) across heterogeneous space [19]. Data of such a broad scope may be difficult to produce within one experiment or study. However, we may approach such a dataset by meta-analysis of a compilation of published estimates of development rate with respect to different environmental factors. In this manner, the phenotype of development rate in response to multiple environmental conditions can be assessed over a wider range of conditions and broader geographical bounds.

*Ae. aegypti* has been well-studied, as it vectors several human pathogens including yellow fever, dengue, and chikungunya, [51,52]. We conducted a meta-analysis of data from studies of the development of *Ae. aegypti* with an aim toward summarizing the impact of multiple environmental conditions on developmental duration, determining the relative importance of these factors, and evaluating their interactions. The conditions evaluated here include temperature, food concentration, food type, larval rearing density, geographic location, and latitude. The linear relationship between development rate and temperature was also evaluated across studies to test the hypothesis that it is a fixed characteristic of the species.

## Methods

### Literature search

For the literature search and meta-analysis we adhered to PRISMA guidelines. We searched online databases for peer-reviewed research papers in December of 2011 pertaining to *Ae. aegypti* development. Of the two forms of *Ae. aegypti*, *Ae. a. formosus*, was not included because of known differences in ecology [53], behavior [54], and spatial distribution [55] with limited gene flow between forms [56]*.* The list of databases searched along with keywords and the number of papers included from each source is summarized in supplementary materials (Table S1). The inclusion criteria were as follows. Studies had to report i) the larval rearing temperature, ii) the development time of mosquitoes from hatch to pupation or hatch to emergence in hours or days (data could be in either tabular or graphical format and graphical data were digitally extracted with PlotDigitizer; copyright 2000–20011, Joseph A. Huwaldt), iii) the number of replicates, and iv) the number of larvae included for each estimate. In order to accomplish a meta-analysis, datasets must have similar experimental designs [57], and we focused on studies that estimated development time with respect to temperature. We made every effort to include as many environmental factors as possible. Whenever reported we also included other methodological information of diet level (in milligrams of food per larva per day), diet type (main ingredient), larval rearing density (number of larvae per milliliter of water), photoperiod, and global position coordinates of the study or, when available and specified, strain origin (Table 1). Studies with transgenic strains were also included with “transgenic” added as another factor. For studies of laboratory strains of mosquitoes, we used the coordinates of the strain’s location of origin. These data were compiled into a Microsoft Excel (Redmond, Washington: Microsoft 2011) spreadsheet and are available in supplementary materials and from the corresponding author.

**Table 1:** **Studies included in the meta-analysis of *Ae. aegypti *** **development**

**Temp**	**Temp gradient**	**Density**	**Density gradient**	**Diet (amt)**	**Diet gradient**	**Diet (type)**	**Photo-period**	**Latitude**	**Author & Year**	**Journal**
**✓**		**✓**	**✓**			**✓**	**✓**	Est.	Bargielowski et al. 2011 [58]	PLoS ONE
**✓**	**✓**	**✓**		**✓**		**✓**	**✓**	**✓**	Farjana et al. 2012 [34]	Med. Vet. Entomol.
**✓**	**✓**	**✓**		**✓**		**✓**		**✓**	Mohammed and Chadee 2011 [59]	Acta Trop.
**✓**	**✓**					**✓**		**✓**	Padmanabha et al. 2011 [60]	Med. Vet. Entomol.
**✓**		**✓**	**✓**			**✓**	**✓**	Est.	Maciá 2009 [61]	Rev. Soc. Entomol. Argent.
**✓**		**✓**	**✓**			**✓**	**✓**	Est.	Reiskind and Lounibus 2009 [62]	Med. Vet. Entomol.
**✓**		**✓**		**✓**		**✓**	**✓**	**✓**	Tejerina et al. 2009 [63]	Acta Trop.
**✓**		**✓**		**✓**		**✓**	**✓**	**✓**	Beserra and Castro 2008 [64]	Neotrop. Entomol.
**✓**		**✓**				**✓**	**✓**	**✓**	Chang et al. 2007 [65]	J. Med. Entomol.
**✓**	**✓**	**✓**		**✓**		**✓**	**✓**	**✓**	Beserra et al. 2006 [66]	Neotrop. Entomol.
**✓**		**✓**		**✓**	**✓**	**✓**	**✓**	**✓**	Arrivillaga and Barrera 2004 [67]	J. Vector. Ecol.
**✓**		**✓**		**✓**	**✓**	**✓**	**✓**	Est.	Bedhomme et al. 2004 [68]	Proc. R. Soc. Lond. B.
**✓**		**✓**		**✓**		**✓**	**✓**	Est.	Irvin et al. 2004 [69]	PNAS
**✓**		**✓**	**✓**	**✓**	**✓**	**✓**		Est.	Agnew et al. 2002 [43]	Ecol. Entomol.
**✓**	**✓**	**✓**		**✓**		**✓**	**✓**	**✓**	Kamimura et al. 2002 [70]	Med. Entomol. Zool.
**✓**		**✓**		**✓**		**✓**	**✓**	Est.	Lounibus et al. 2002 [71]	J. Vector. Ecol.
**✓**	**✓**	**✓**				**✓**		Est.	Tsuda and Takagi 2001 [72]	Environ. Entomol.
**✓**	**✓**	**✓**		**✓**		**✓**	**✓**	**✓**	Tun-Lin et al. 2000 [73]	Med. Vet. Entomol.
**✓**								Est.	Costero et al. 1999 [74]	J. Med. Entomol.
**✓**		**✓**	**✓**			**✓**	**✓**	Est.	Silva and Silva 1999 [75]	Rev. Soc. Bras. Med. Trop.
**✓**	**✓**							Est.	Thu et al. 1998 [76]	SE Asian Trop. Med.
**✓**	**✓**	**✓**		**✓**		**✓**	**✓**	Est.	Becnel and Undeen 1992 [77]	J. Invertebr. Pathol.
**✓**	**✓**	**✓**				**✓**	**✓**	Est.	Rueda et al. 1990 [78]	J. Med. Entomol.
**✓**		**✓**		**✓**		**✓**	**✓**	Est.	Ho et al. 1989 [79]	J. Med. Entomol.
**✓**		**✓**	**✓**	**✓**	**✓**	**✓**	**✓**	Est.	Russell 1986 [80]	Aust. J. Zool.
**✓**						**✓**	**✓**	Est.	Soekiman et al. 1984 [81]	ICMR Ann.
**✓**		**✓**	**✓**	**✓**		**✓**	**✓**		Dye 1982 [82]	Ecol. Entomol.
**✓**		**✓**		**✓**		**✓**		Est.	Saul et al. 1980 [83]	Am. Midl. Nat.
**✓**	**✓**					**✓**		Est.	Gilpin and McClelland 1979 [84]	Fortschr. Zool.
**✓**		**✓**				**✓**		Est.	Dadd et al. 1977 [85]	Mosq. News
**✓**	**✓**	**✓**						Est.	Lachmajer and Hien 1975 [86]	Inst.t Med. Morskiej I Trop.
**✓**		**✓**				**✓**		Est.	Ameen and Moizuddin 1973 [87]	Dacca Univ. Stud.
**✓**		**✓**		**✓**		**✓**		Est.	Moore and Whitacre 1972 [88]	Ann. Entomol. Soc. Am.
**✓**								Est.	Southwood et al. 1972 [89]	Bull. World Health Organ.
**✓**		**✓**						Est.	Rosay 1972 [90]	Mosq. News
**✓**									Nayar 1970 [91]	J. Med. Entomol.
**✓**	**✓**					**✓**		Est.	McCray et al. 1970 [92]	J. Invertebr. Pathol.
**✓**		**✓**				**✓**		Est.	Keirans 1969 [93]	Mosq. News
**✓**		**✓**	**✓**	**✓**	**✓**	**✓**	**✓**	Est.	Moore and Fisher 1969 [94]	Ann. Entomol. Soc. Am.
**✓**		**✓**		**✓**	**✓**	**✓**		Est.	Peters et al. 1969 [95]	Mosq. News
**✓**		**✓**				**✓**		Est.	Brust 1968 [96]	J. Econ. Entomol.
**✓**	**✓**	**✓**				**✓**		Est.	Keirans and Fay 1968 [97]	Mosq. News
**✓**	**✓**	**✓**	**✓**	**✓**	**✓**	**✓**		Est.	Wada 1965 [98]	Quaestiones entomologicae
**✓**		**✓**		**✓**		**✓**		Est.	Lea 1963 [99]	J. Insect. Physiol.
**✓**	**✓**	**✓**		**✓**				**✓**	Ofuji 1963 [100]	B. Res Inst. Endem. Nagasaki Univ.
									Christophers 1960 [101]	Cambridge University Press
**✓**	**✓**	**✓**				**✓**		Est.	Bar-Zeev 1958 [102]	B. Entomol. Res.
**✓**	**✓**								Headlee 1940 [103]	J. Econ. Entomol.
**✓**	**✓**					**✓**			Headlee 1941 [104]	J. Econ. Entomol.

Check marks indicate studies that have reported at least one value of the environmental conditions listed including temperature, diet (mg/larva/day), density (larvae/mL), or photoperiod. Gradient columns indicate whether the study considered three or more levels of the environmental condition. Latitude of origin was either reported (check mark) or estimated (Est.) based on the city of origin of the mosquito strain. Studies that considered transgenic strains are indicated in bold. Development rate estimates for transgenic strains were not included in the meta-analysis. A full bibliography is available in Additional file 1: Table S2.

### Meta-analysis

We used two meta-analytic approaches for these data. In our first approach we evaluated estimates of development time from hatch to pupation and development time from hatch to emergence using a mixed linear regression model [105] “nlme” [106] implemented in R statistical software v3.0.2 [107]. These two dependent variables were analyzed separately. Factors evaluated included temperature, larval rearing density, diet level (mg/larvae/day), latitude of strain origin, photoperiod, and publication. For a study to be included in the mixed linear regression model at least one environmental factor had to be reported along with the estimate of development time (i.e. at least one temperature, larval rearing density, or diet level). The variable of sex was not considered for hatch to emergence in this portion of the analysis as many studies reported values for only females or did not report sex at all. Publication author was considered a random factor in our analysis as our primary interest was the in the effects of other variables across studies. Parameters were eliminated using backward model selection and a minimization of the Akaike Information Criterion (AIC) and BIC (Bayesian Information Criterion). Both criteria impose a penalty for increasing the number of parameters in a model. A model with ΔAIC = 2 and ΔBIC = 2 or more units lower than any other model was considered the best [105].

In our second approach, we focused analysis on a temperature range for which development rate (1/development time) can be well approximated with a linear model. Development rates in *Ae. aegypti* are well approximated by a linear model within the temperature range from 14 **-** 31°C [84]. The linear model is described with the following equation,

$$y=B_{1}x+B_{0},$$

where y = 1/development rate, and y is regressed on temperature, x. The parameter *B*_*0*_ represents the developmental zero and *B*_*1*_ is a constant for the cumulative effective of temperature, generally reported as *K*[108,109]. When parameter estimates were not directly reported, linear models were run in the open source package R version 2.14.0 (R Development Core Team 2012). Linear models in this second meta-analytic approach were only conducted on data from studies that estimated development rate over three or more temperatures in order to allow for a regression analysis. For meta-analysis, parameter estimates of *B*_*1*_ and *B*_*0*_ were each used as effect measures, and were weighted by the number of replicates per experiment in a study. We tested the hypotheses that cumulative effect of temperature (*K*) and developmental zero (*t*) are constant properties of a mosquito strain using a test of total heterogeneity, Q_T_, with Hedge’s estimator, a standardized difference method for comparing effect measures [57,110]. Next, we used a linear mixed effects model to determine the variables that best explained this heterogeneity including publication, diet, larval rearing density, and latitude of strain origin. We then tested for residual heterogeneity, Q_E_[57,110]. For this portion of the analysis we were able to include the variable of sex due to greater reporting in this subset of studies. Sex was considered with three categories: male, female, and both.

## Results

**Figure 1:**
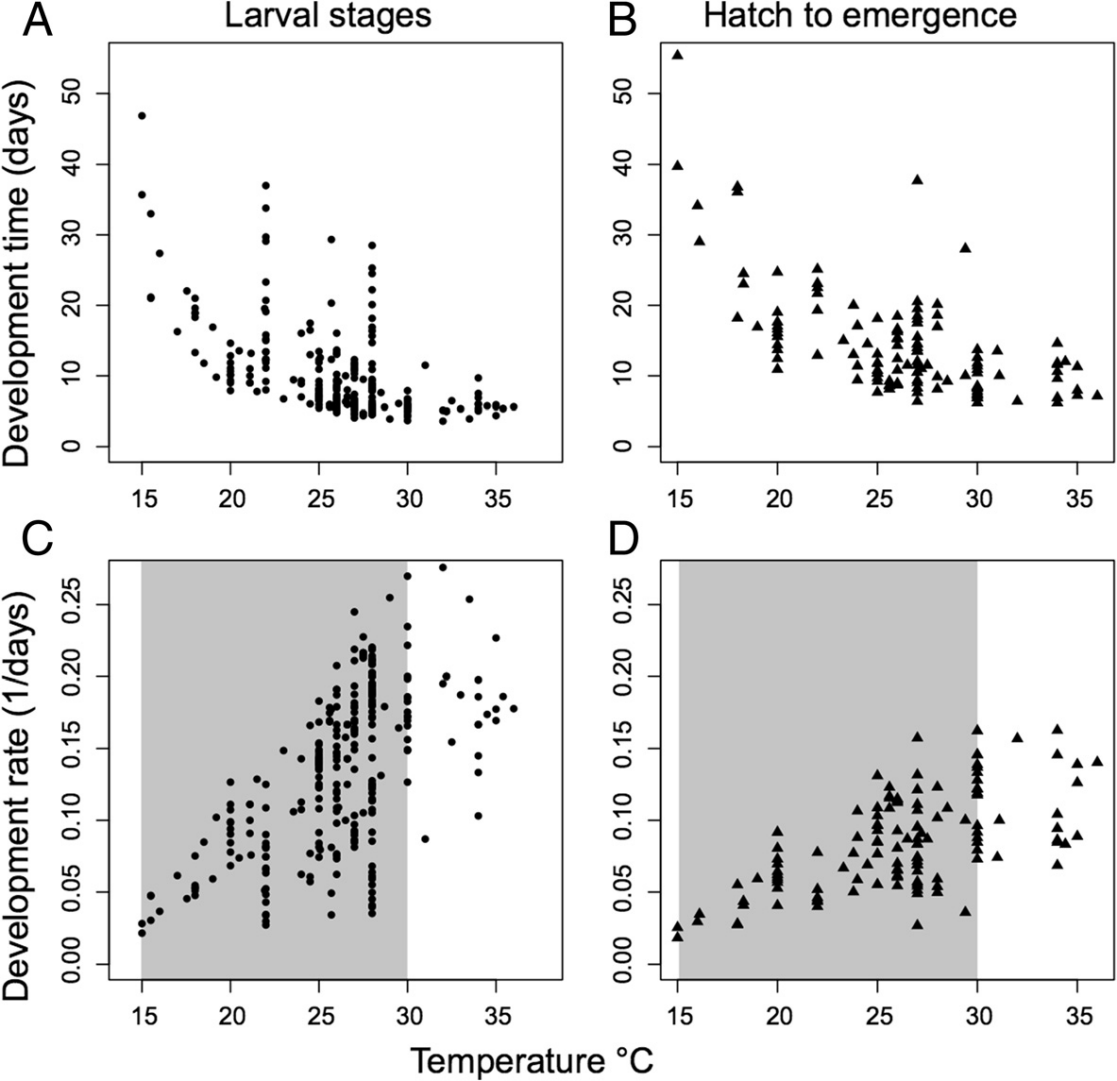
**Compiled dataset of development time (days) and development rate (1/days) plotted against temperature for hatch to pupation, i.e. larval stages (A and C, respectively), and hatch to emergence (B and D respectively).** Shaded gray bars show the subset of data used for linear models of development rate.

**Figure 2:**
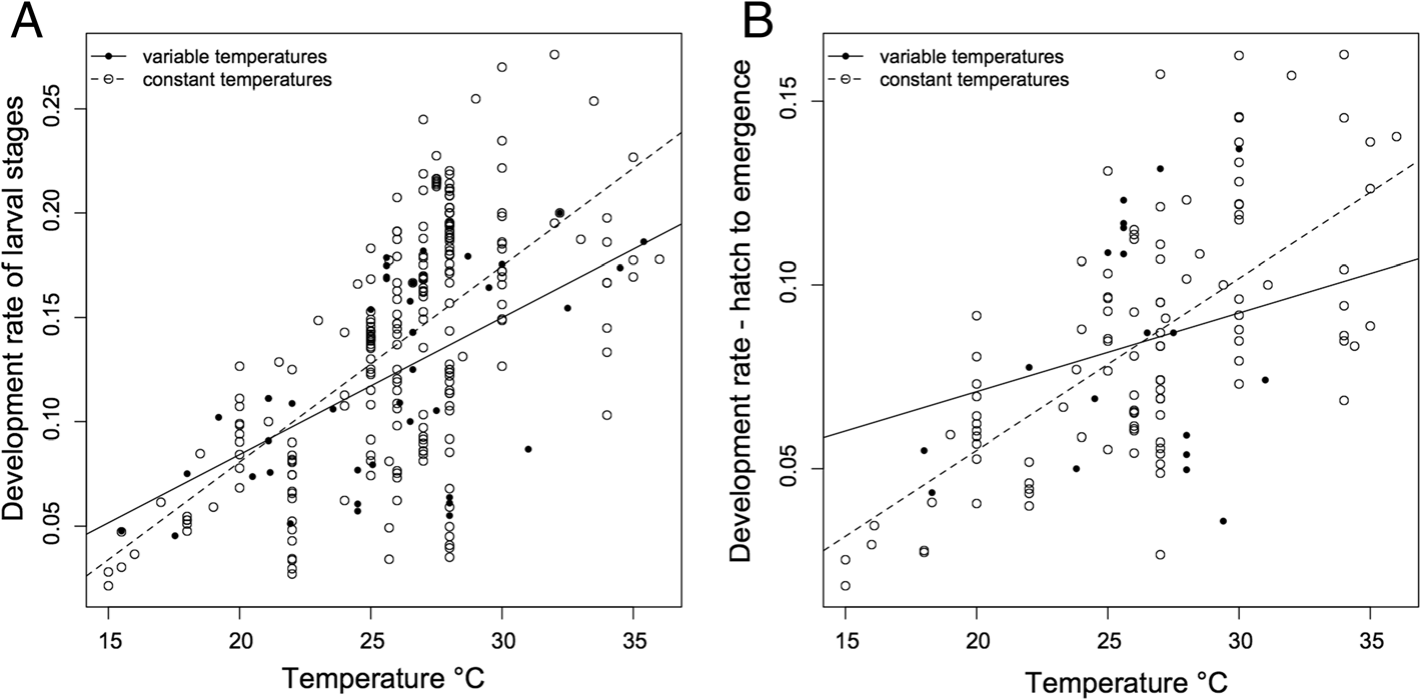
**Development rate (inverse development time) estimates for (A) hatch to pupation, i.e. larval stages, and (B) hatch to emergence plotted against temperature.** Character shape represents whether larvae were reared in constant or variable temperatures. Line type corresponds with character shape and lines indicate linear regression of development rate and temperature for constant and variable temperatures.

Based on a literature search of 11 online databases using search terms including *Aedes aegypti*, temperature, diet, larval rearing density, and development rate, we found 27,559 articles, from which 48 journal publications and one book chapter fit the inclusion criteria (Table 1). From these, data on development rate were compiled for 66 populations of *Ae. aegypti* (references in Additional file 1: Table S2; dataset available upon request) spanning approximately 87° of latitude (Figure 1). Among these studies, 39% evaluated temperature across a gradient of 2 or more levels, and 77% of all reported one intraspecific rearing density whereas 18% considered larval rearing density gradients. Many studies reported food added *ad libitum,* but among the subset of studies that reported diet values, 25% examined diet gradients. Photoperiod was reported in 45% of studies. Some studies were laboratory based and others were field-based or under semi-natural conditions. This facilitated the comparison of constant versus variable temperatures on development rate (Figure 2).

The type of diet was reported for 42 of 49 experiments, and of these studies 32 had a unique diet composition. Diets shared across multiple studies included brewer’s yeast and Tetramin**®** Fish Food. Unique diets were combinations of these and various other sources including, but not limited to, rabbit food, dog food, pig chow, pig liver powder, beef liver powder, bacterial infusions, detritus, and unspecified larval broth. Inclusion of diet type led to over-parameterization of models and was dropped from the analysis as a factor due to the number of unique types.

Development time of larval stages, development time from hatch to emergence, and percent survival were compiled into a dataset for the first meta-analytic approach (Additional file 1: Table S2). Inclusion required an estimate of development rate of *Ae. aegypti* under at least one value of temperature, larval rearing density, or diet. The compiled dataset had 283 estimates of development time from hatch to pupation and 127 from hatch to emergence (Figure 1, panels A and B). Temperatures ranged from 14–37.8°C. Development times were not normally distributed for larval stages (Shapiro-Wilk test, W = 0.727, p < 0.0001) or from hatch to emergence (W = 0.7942, p < 0.0001), and therefore estimates were transformed into development rate in the form of the inverse of development time. Development rates were normally distributed for larval stages (W = 0.9797, p > 0.08) and hatch to emergence (W = 0.9532, p > 0.1). Development rate showed a significant positive association with rearing temperature across all studies for larval stages (*B*_*1*_ = 0.008913, t_281_ = 13.50, p < 0.0001, *R*^*2*^ = 0.3782; Figure 1, panel C). Similarly, the development rate from hatch to emergence is significantly associated with temperature (*B*_*1*_ = 0.0045222, t_125_ = 8.725, p < 0.0001, *R*^*2*^ = 0.3862; Figure 1, panel D).

For better approximation with a linear model we used a subset of the compiled data over the temperature range of 14 **-** 31°C resulting in 262 estimates for larval stages and 110 for hatch to emergence. This data subset restricted only the upper boundary of development rate estimates, above which a linear model is no longer a good approximation (Figure 1) [84]. The full GLMM model for development rates included fixed factors of temperature, photoperiod, diet, larval rearing density, and a dummy variable of temperature variability (constant or variable temperature). Estimates under constant temperatures came from laboratory studies. Estimates under variable temperatures came from both field studies in natural or semi-natural conditions and laboratory studies with fluctuating temperature schemes accomplished using environmental chambers. Temperature fluctuations imposed in laboratory studies differed in magnitude, duration, and the life stage at which mosquitoes were exposed. To broadly assess the difference between constant and variable temperatures we created the dummy variable of temperature variability. Random factors included latitude and publication. Based on the minimum AIC and BIC, the best model for development rate from hatch to pupation included the fixed factor of temperature and the random factor of publication (Table 2). Similarly the best model for the development rate from hatch to emergence included only temperature as a fixed factor and the random factor of publication (Table 3).

**Table 2:** **Linear mixed effects model selection of *Ae. aegypti *** **development rate from hatch to pupation**

**Fixed factor**	**Random factor**	**AIC**	**Δ AIC**	**BIC**	**Δ BIC**
T, Ph, D, Dt, EV	Author, Lat	-36.74	436.16	-15.53	443.07
T, Ph, D, Dt, EV	Lat	-35.65	437.25	-16.79	441.81
T, Ph, D, Dt, EV	Author	-118.3	354.6	-96.28	362.32
T, Ph, D, Dt	Author	-124.9	348	-105.6	353
T, Ph, Dt, EV	Author	-127.4	345.5	-108.2	350.4
T, Ph, D, EV	Author	-196.5	276.4	-175.2	283.4
T, D, Dt, EV	Author	-215.4	257.5	-193.9	264.7
Ph, D, Dt, EV	Author	39.35	512.25	58.62	517.22
T, D, Dt	Author	-222.8	250.1	-204.5	254.1
T, Dt, EV	Author	-224	248.9	-205.6	253
T, D, EV	Author	-395.2	77.7	-374.5	84.1
D, Dt, EV	Author	1.712	474.612	20.09	478.69
T, D	Author	-403.5	69.4	-386.2	72.4
T, EV	Author	-466.8	6.1	-448.9	9.7
D, EV	Author	-96.4	376.5	-79.1	379.5
EV	Author	-131.6	341.3	-117.3	341.3
**T**	**Author**	**-472.9**	**0**	**-458.6**	**0**

Fixed factors considered were temperature (T), photoperiod (Ph), density in larvae/mL (D), diet in mg/larva/day (Dt), and environmental variability (EV). Environmental variability represents constant versus variable temperatures. Random factors included study author (Author) and latitude of origin for the *Ae. aegypti* study strain. AIC and BIC stand for Akaike and Bayes Information Criterion respectively. ∆ represents the difference with respect to the minimum value. The best model with minimum values for each selection criterion is bolded. The AIC and BIC have negative values because the models had positive log-likelihoods, which occur because the probability densities evaluated at the observations are below 1, which produces a negative logarithm. ∆AIC and ∆BIC show differences with respect to the model that minimized each information criterion.

**Table 3:** **Linear mixed effects model selection of *Ae. aegypti *** **development rate from hatch to emergence**

**Fixed factor**	**Random factor**	**AIC**	**Δ AIC**	**BIC**	**Δ BIC**
T, Ph, D, Dt, EV	Author, Lat	-71.58	131.32	-51.24	140.86
T, Ph, D, Dt, EV	Lat	-69.23	133.67	-51.25	140.85
T, Ph, D, Dt, EV	Author	-73.48	129.42	-55.49	136.61
T, Ph, D, Dt	Author	-76.71	126.19	-60.97	131.13
T, Ph, Dt, EV	Author	-78.76	124.14	-63.02	129.08
T, Ph, D, EV	Author	-89.58	113.32	-73.65	118.45
T, D, Dt, EV	Author	-98.46	104.44	-82.06	110.04
Ph, D, Dt, EV	Author	24.1	227	39.84	231.94
T, D, Dt	Author	-105.3	97.6	-91.2	100.9
T, Dt, EV	Author	-106.5	96.4	-92.4	99.7
T, D, EV	Author	-140.2	62.7	-125	67.1
D, Dt, EV	Author	9.06	211.96	23.13	215.23
T, D	Author	-147	55.9	-134.3	57.8
T, EV	Author	-195	7.9	-181.5	10.6
D, EV	Author	-12.11	190.79	0.55	192.65
EV	Author	-43.19	159.71	-32.39	159.71
**T**	**Author**	**-202.9**	**0**	**-192.1**	**0**

Fixed factors considered were temperature (T), photoperiod (Ph), density in larvae/mL (D), diet in mg/larva/day (Dt), and environmental variability (EV). Environmental variability represents constant versus variable temperatures. Random factors included study author (Author) and latitude of origin for the *Ae. aegypti* study strain. The best model with minimum values for each selection criterion is bolded.

For the second meta-analytic approach, inclusion required estimation of development rate for at least three temperatures in one experiment. The regression parameters for development rate on temperature are reported in supplementary tables (Additional file 1: Tables S3 and S4). The estimates of the developmental zero (t) and degree-day model constant (K) are calculated and listed for each study for both hatch to emergence (Table 4) and hatch to pupation (Table 5). The literature search yielded 23 experiments meeting the criteria with the dependent variable development rate from hatch to emergence. The literature search yielded 20 experiments meeting the criteria for development rate from hatch to pupation. Results of experiments conflicted regarding the significance of the relationship between temperature and development rate. For example, considered separately, many of the studies did not show a significant, positive linear relationship between temperature and development rate (Tables 4 and 5). Of the 23 studies measuring hatch to emergence, 10 did not find a significant linear association. Similarly, 7 of 20 studies did not show a significant relationship for development rate from hatch to pupation and temperature. However, these data combined demonstrated an overall significantly positive association (Figure 2).

**Table 4:** Studies that estimated development rate to adult emergence over three or more temperatures

**Study**	**Latitude**	**Sex**	**t (°C)**	**K**	**n**	**r**^**2**^	**p-value**	
Bar-Zeev 1958 [102]	31.0461	F	12.83	121.86	100	0.9959	6.21E-06	***
Beserra et al. 2006 [66]	-7.4908	C	13.35	186.74	120	0.9874	0.00634	*
Beserra et al. 2006 [66]	-6.38	C	9.40	280.23	120	0.9962	0.03915	*
Beserra et al. 2006 [66]	-7.2256	C	8.42	243.21	120	0.8418	0.2604	
Beserra et al. 2006 [66]	-7.3	C	13.63	173.32	120	0.9949	0.002563	**
Beserra et al. 2006 [66]	-6.9669	C	18.35	102.82	120	0.9644	0.1209	
Farjana et al. 2012 [34]	-3.3439	F	9.95	257.90	100	0.981	0.08805	
Farjana et al. 2012 [34]	-3.3439	F	11.44	158.13	100	0.9403	0.1572	
Farjana et al. 2012 [34]	-3.3439	M	9.95	209.14	100	0.9882	0.06917	
Farjana et al. 2012 [34]	-3.3439	M	11.69	137.59	100	0.9318	0.1682	
Headlee 1941 [104]	40.486217	C	10.21	187.68	200	0.9828	0.0838	
Headlee 1940 [103]	40.486217	C	8.38	219.88	200	0.9858	0.0007197	***
Kamimura et al. 2002 [70]	24.8934	F	9.93	162.44	50	0.9902	0.06328	
Kamimura et al. 2002 [70]	-7.2653	F	10.68	151.77	50	0.9985	0.02504	*
Kamimura et al. 2002 [70]	-9.2628	F	11.38	144.78	50	0.9472	0.1476	
Kamimura et al. 2002 [70]	24.8934	M	8.19	176.84	50	0.9931	0.05285	*
Kamimura et al. 2002 [70]	-7.2653	M	10.10	148.90	50	0.9977	0.03039	*
Kamimura et al. 2002 [70]	-9.2628	M	9.09	163.45	50	0.9142	0.1893	
Lachmajer & Hien 1975 [86]	14.0583	C	6.85	141.43	6300	0.9958	0.04125	*
Ofuji 1963 [100]	32.2	F	10.76	133.80	20	0.96	0.00344	**
Ofuji 1963 [100]	32.2	M	10.45	129.82	20	0.9514	0.004608	**
Rueda et al. 1990 [78]	35.7721	C	11.17	129.35	20	0.8669	0.006966	*
Tun-Lin et al. 2000 [73]	-10.58	C	46.31	332.82	200	0.8497	0.02594	*

Developmental zero (t) and linearized degree day model constant (K) are listed along with the correlation coefficient and p-value of the linear regression between temperature and development rate. Level of significance is indicated by the number of asterisks (*< 0.01; **< 0.001; ***< 0.0001). Sex is listed as C if values represent a combination of males and females.

**Table 5:** Studies that estimated development rate to pupation over three or more temperatures

**Study**	**Latitude**	**t (°C)**	**K**	**n**	**r**	**p-value**	
Bar-Zeev 1958 [102]	31.0461	-14.21	86.22	100	0.9975	0.001269	**
Becnel & Undeen 1992 [77]	15.87	1.13	185.46	250	0.883	0.2222	
Beserra et al. 2006 [66]	-7.4908	-9.91	148.46	120	0.8963	0.01464	*
Beserra et al. 2006 [66]	-6.38	-7.75	187.97	120	0.9758	0.001609	**
Beserra et al. 2006 [66]	-7.2256	-9.41	130.57	120	0.8663	0.02164	*
Beserra et al. 2006 [66]	-7.3	-4.37	200.88	120	0.4404	0.222	
Beserra et al. 2006 [66]	-6.9669	-12.56	114.48	120	0.8652	0.02193	*
Gilpin & McClelland 1979 [84]	-10.9491	-10.81	82.27	300	0.8875	4.80E-07	***
Kamimura et al. 2002 [70]	24.8934	-1.62	28.64	50	0.9634	0.1225	
Kamimura et al. 2002 [70]	-7.2653	-9.70	122.78	50	0.9995	0.01357	*
Kamimura et al. 2002 [70]	-9.2628	-9.89	122.34	50	0.9035	0.2011	
Keirans & Fay 1968 [97]	18.2208	-10.79	102.18	50	0.9729	6.26E-06	***
Lachmajer & Hien 1975 [86]	14.0583	-10.00	112.68	6300	0.9598	0.1286	
Mohammed & Chadee 2011 [59]	10.6389	69.92	365.94	600	0.002094	0.9069	
Ofuji 1963 [100]	32.2	-9.70	105.79	20	0.9095	0.01189	*
Padmanabha et al. 2011 [36]	10.9861	-9.09	100.97	160	0.9644	0.0004806	***
Rueda et al. 1990 [78]	35.7721	-10.65	101.43	20	0.7966	0.01671	*
Thu et al. 1998 [76]	21.914	76.45	1124.99	100	0.0356	0.8113	
Tsuda & Takagi 2001 [72]	19.5177	-10.40	153.68	50	0.6096	0.03826	*
Tun-Lin et al. 2000 [73]	-10.58	-36.15	727.80	200	0.887	0.01671	*

Developmental zero (t) and linearized degree day model constant (K) are listed along with the correlation coefficient and p-value of the linear regression between temperature and development rate. Level of significance is indicated by the number of asterisks (*< 0.01; **< 0.001; ***< 0.0001).

The linear association between development rate and temperature had significant heterogeneity for both hatch to pupation (Q_T_ = 242.4396, p < 0.0001) and hatch to emergence (Q_T_ = 403.5, p < 0.0001). A linear mixed effects model was used to determine what other environmental factors might explain the heterogeneity in this relationship. Additional factors considered were initial larval rearing density, diet level (mg/larva/day), strain origin, latitude, and publication author. The model including only temperature as a fixed factor and the random factor of publication author best explained the heterogeneity in slope estimates for both the pupation group and emergence group. Once publication was included in the model, the test of residual heterogeneity was no longer significant for hatch to pupation (Q_E_ = 4.8582, p < 0.3022) or hatch to emergence (Q_E_ = 2.23, p < 0.8971). Similarly, the developmental zero was significantly heterogeneous for both the hatch to pupation development rate (Q_T_ = 92.3908, p < 0.0001) and hatch to emergence (Q_T_ = 675.6708, p < 0.0001). Once temperature had been considered, the residual heterogeneity in the developmental zero was explained by publication author such that the test for residual heterogeneity was no longer significant (hatch to pupation: Q_E_ = 2.2802, p < 0.6844; hatch to emergence: Q_E_ = 1.0234, p < 0.9847).

**Figure 3:**
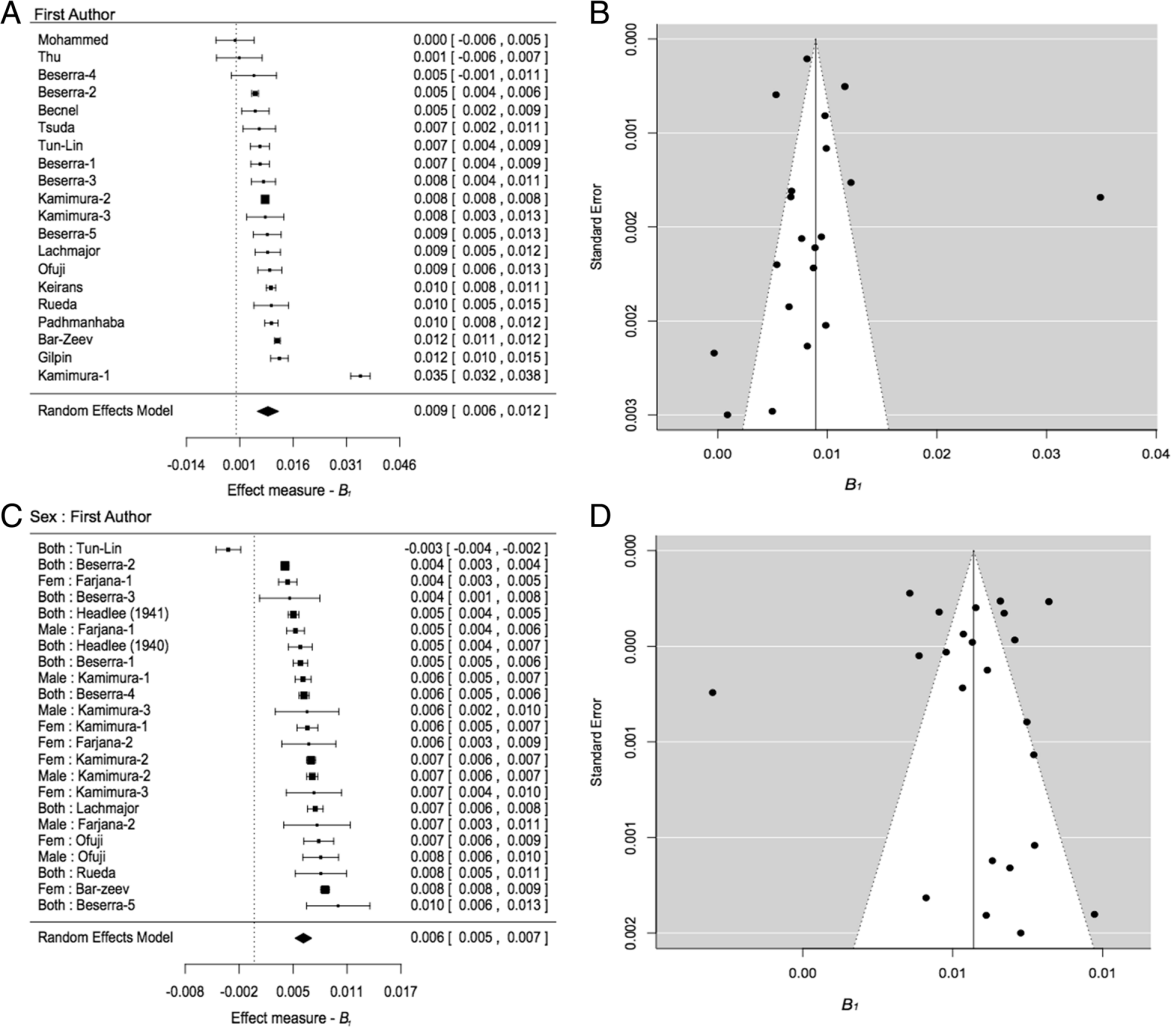
**Meta-analysis of the effect of temperature, i.e. *B***_***1 ***_***- *the slope of the regression of temperature and development rate. (A)** Forest plot for development rates of hatch to pupation, i.e. larval stages. **(B)** Funnel plot corresponding to plot **(A)**. **(C)** Forest plot for development rates from hatch to emergence. **(D)** Funnel plot corresponding to plot **(C)**. The weight of the study is indicated by the size of the square and the diamond indicates the overall effect estimate from the random effects model. First authors are listed on the left of the forest plots and, when applicable, the strain identifier is listed by number (for full references see Additional file 1: Table S2). Squares represent effect estimates of individual studies. Square size represents the weight given to the study in the meta-analysis, and the horizontal lines represent 95% confidence intervals. Estimated values and confidence intervals are written to the right of the plot. In the funnel plots, points represent the residuals of the model presented in the corresponding forest plot and their associated standard error. When the residuals fit within the light cone, it implies that heterogeneity in the main effect is successfully accounted by the model.

Asymmetry was apparent when plotting effect measures against study size in funnel plots (Figure 3). In the absence of systematic heterogeneity, points should fall within the range indicated by the inverted cone in funnel plots. Asymmetry may be a result of publication bias or systematic heterogeneity. With the inclusion of publication author as a random effect in the model, the asymmetry was no longer evident and the funnel plots no longer indicated heterogeneity for hatch to emergence or hatch to pupation (Additional file 2: Figure S1 and Additional file 3: Figure S2).

**Figure 4:**
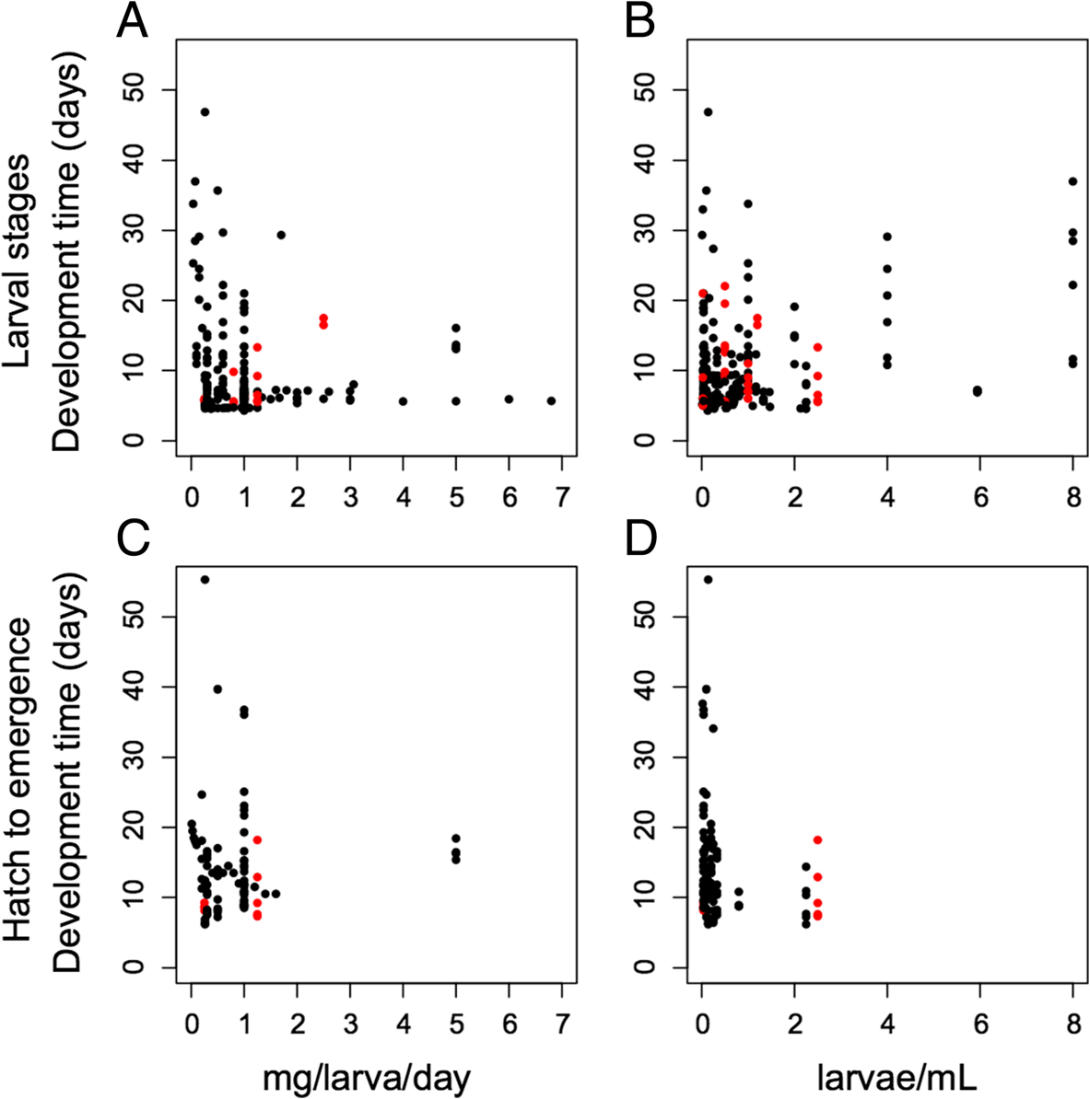
**Development time of hatch to pupation, i.e. larval stages, compared to diet (A) and density (B), and development time from hatch to emergence compared to diet (C) and density (D).** Character color indicates laboratory (black) and field studies (red).

The range of diets considered across all studies was 0.01 mg/larva/day to 435.2 mg/larva/day. However, 96.6% of studies used values within the range of 0.01 mg/larva/day to 6.8 mg/larva/day. Comparisons of diet level with development rate are shown in Figure 4, panels A and C. The larval rearing density considered across the studies ranged from 0.01 larvae/mL to 8 larvae/mL, and comparisons with development rate are shown in Figure 4, panels B and D. Approximately 69% of larval rearing density levels used by studies in the meta-analysis fell between 0.1 larva/mL and 1 larva/mL.

## Discussion

We hypothesized, first, that development rate is significantly influenced by several environmental factors and that the interaction of these factors is an important predictor of development rate variation. The results of both meta-analytic approaches suggest that temperature is the main fixed factor driving development rate, to the exclusion of other factors of known importance such as diet and density. This bolsters the contention that temperature is the most important ecological determinant and, when modeling development, sufficient to predict development rate [111]. When larvae experience nutritional deprivation or high densities, this can dampen [112] or exacerbate [36] the impacts of temperature. Thus, while research suggests that diet [73,113] and larval rearing density [84,114] do matter, these results underscore that they should not be considered to the exclusion of temperature. Based on model selection, the relative importance of these factors can be ranked as temperature followed by temperature variability, larval rearing density, then diet, and lastly photoperiod (Table 2, Table 3). The relative importance of factors is consistent between the periods of hatch to pupation and hatch to emergence. While this analysis shows other variables such as latitude were not significant in explaining development rate variation, they may impact other important life history traits including survival, body size, fecundity [33], and morphology [115].

The relationship between temperature and development rate is linear within a median temperature range [116-119], and features of this linear relationship, such as slope and intercept, have biological interpretations. The slope of this relationship is considered the cumulative effect of temperature on the rate of development, and the intercept can be interpreted as the theoretical temperature at which development can no longer occur [5,109], also called the developmental zero. Although at extremes of low temperature the development curve is non-linear, the linear portion is extrapolated to the intersection with the temperature axis to estimate the developmental zero [120]. This extrapolation based on slope may, in part, explain the large variation in the estimates reported in Tables 4 and 5. This may also explain estimates that were less than zero, which is biologically implausible. Meta-analysis these parameters across many studies allows for outliers to be more easily identified.

Despite these limitations, the developmental zero is often considered a fixed characteristic of a species for the purposes of modeling and predicting population abundance [39,109,121,122]. Thus, we also sought to test the hypothesis that the effect of temperature and the developmental zero are fixed characteristics of *Ae. aegypti* strains*.* While the meta-analytic results are consistent with a positive, linear relationship between temperature and development rate, tests for heterogeneity suggest a significant amount of variation in response to temperatures within this range. These data do not support the hypothesis that the developmental zero and the effect of temperature are fixed constants. Both the effect of temperature and the developmental zero are heterogeneous across studies considered in the meta-analysis. These results have implications for the modeling of development rate as well as population abundance, which often relies on development times of larval populations [22,123]. These compiled data may be used as the basis for modeling these parameters as a distribution rather than choosing one value from a single study. Variation in development time (i.e. the inverse of development rate) has been modeled as a continuous random variable with a distribution of frequencies, such as the normal distribution [124] or with a heterogeneity factor [125]. Other modeling approaches to incorporate development rate variation stochastically by treating development rate as a random variable dependent on the variability in the level of catalytic enzymes [126-128], positing a biophysical basis for variability.

There are several hypotheses to address why the response to temperature may be heterogeneous. Our results indicate that factors of larval rearing density, diet, latitude, and photoperiod were not factors that could explain heterogeneity of the effect of temperature. A limitation of this analysis was the narrow range of reported values of diet and initial larval rearing density. While many studies reported at least one level of different factors such as temperature, diet, and larval rearing density, few studies in *Ae. aegypti* examined development across gradients of multiple environmental conditions. Such experiments are needed in order to establish the relative importance of environmental factors in the variation of development rates. Assessing the impact of varied environmental conditions on the developmental phenotypes of mosquito larvae can be complex with interactive effects [18,24,129]. For example, Padhmanhaba *et al.* 2011 [36] show that increased the rearing temperature for starved *Ae. aegypti* larvae impacts development rate, and this impact changes depending on the larval stage and the temperature.

Publication author was adequate to explain heterogeneity in the effect of temperature on development rates. It is difficult to identify the aspects of this factor to describe its significance in explaining development rate variation. We evaluated the dichotomy of laboratory versus field experiments, which generally corresponded to constant versus variable temperatures. Mosquito response to variable rather than constant temperatures has been a recent focus both for life history traits and vectorial capacity [123,130-134]. Variable temperatures have been shown to increase [135], decrease [118], and have no impact [136] on development rates of mosquitoes and other insects. Inconsistency in the relationship between temperature and development rate has been attributed to field conditions versus laboratory conditions [36]. To test this, we compared development rates estimated under constant versus variable temperature conditions*,* which corresponded to laboratory versus field conditions. This comparison showed no significant difference overall in the relationship between development rate and temperature based on temperature variability for either larval stages or to hatch to emergence (Figure 2). This finding is consistent with recent reports that *Ae. aegypti* life-history traits depend not only on variability but also the magnitude of temperature fluctuations [134].

The factor of publication may be a proxy for methodological differences such as diet composition (i.e. ingredients of diet). Of the 49 studies, almost all reported information on diet composition. However, few used the same diet preparations, and this prevented this factor from being included in meta-analysis. Some diets were created from detritus of the larval habitat in order to mimic natural conditions [36,61,137] or incorporated detritus [62]. The majority however provided no explanation for the choice of diets. Diet choice can influence development rate as well as interspecific larval competition [138,139] and adult wing length [60]. To facilitate comparison of larval performance across populations, these findings support a need for standardization of diet composition for laboratory colonies*.* This is especially important in the context of transgenics. Our literature search yielded only two studies with estimating development rate of *Ae. aegypti* transgenic strains. The low sample size impeded statistical comparison of transgenic versus wild-type development rate estimates, leading to singularity errors in the linear mixed effects modeling. Future comparisons of transgenic and wild strains in other important life-history traits such as body size, fecundity, and longevity may also be informative. Estimating and evaluating life-history traits across different environmental conditions is critical to provide a basis for comparison between wild and transgenic strains and may guide future transgenic release programs [29,58,69].

Other factors not considered in this analysis may also impact the effect of temperature, and perhaps contribute to heterogeneity. Examples include genetic variation, microbial symbiotic partners, and maternal effects. Population differences in larval survival and body size in response to different temperatures have been demonstrated in other insects [140] but such differences have also been attributed to adaptive phenotypic plasticity through a hormonal cascade that stops growth [141]. Inclusion of latitude as a variable was one proxy for comparing populations broadly. Latitude has been suggested as a potential gradient for local adaptation to thermal stress in mosquitoes [142]. However, our results suggest latitude does not explain heterogeneity of the effect of temperature. The strain origin/study location was included as a random effect as another indirect proxy for genetic differences in population, but we found no associations with strain origin. There is evidence of genetic structure across geographic space [143] and seasons [144], but examples of strong local adaptation in development rate is lacking in *Ae. aegypti* populations [123]. Richardson et al. [123] suggested that the lack of strong local adaptation may be evidence of a limited capacity to evolve in response to thermal stress. More studies are needed to evaluate the potential for adaptive phenotypic plasticity in response to temperature in *Ae. aegpyti* that could explain the heterogeneity of responses characterized here. Further, in natural conditions other ecological factors not considered here such as interspecific competition, such as between *Ae. aegypti and Ae. albopictus*[34,137]*,* and predation [117] may impact development rate and warrant further investigation.

Recent work compares life-history traits such as body size and fecundity across multiple environmental conditions [145,146]. More empirical estimates of these traits across environments have been recently made available since the preparation of this work [123,134,147,148], a limitation of conducting a meta-analysis in a rapidly developing field of research. Recent advances suggest variation in these traits has been attributed to responses to environmental conditions during development [20,130,149] as well as adaptive genetic responses due to selection at different temperatures [7,150]. Developmental life-history traits are of particular epidemiological importance for arboviral disease dynamics as they have been associated with critical aspects of vectorial capacity such as changes in bite rate, dispersal [151] and virus infection and dissemination [152].

## Conclusion

Beyond utility for vector population control, development rate estimates may be useful for modeling and understanding disease transmission. There is evidence that larval environment impacts adult dispersion of *Ae. aegpyti*[153] as well as arbovirus infection [154]. Depinay et al. 2004 [155] have demonstrated improved predictive power for malaria transmission dynamics when using vector population parameters including life-history traits of anopheline mosquitoes. Meta-analysis confirms that temperature is the most important ecological determinant of development rate in *Ae. aegypti* but that the effect is heterogeneous. Ignoring the heterogeneity in response to temperature may be problematic for using development rate estimates to model vector populations and predicting the impact of temperature on vector-borne disease transmission.

## Competing interests

All authors have read and understood the BMC Ecology policy on declaration of competing interests and declare: no financial relationships with any organizations that might have an interest in the submitted work; no other relationships or activities that could appear to have influenced the submitted work; no patents relating to the content of the manuscript; no financial or non-financial competing interests.

## Authors’ contributions

JC conceived the study, acquired data through a literature search, analyzed and interpreted data, and drafted manuscript. MQB acquired data through literature search, interpreted data, and revised manuscript critically for important intellectual content. Both authors have read and approved the final manuscript.

## Supplementary Material

Additional file 1: Table S1 Online database searched in December 2011 for research papers pertaining to *Aedes aegypti* development rate estimates under various environmental conditions including temperature, diet and intraspecific rearing density. Databases are ordered based on specificity to mosquito literature from broad to specific. **Table S2.** Full bibliography for the 65 studies included in the factors influencing development rate and survival of *Aedes aegypti*. **Table S3.** Linear regression parameter estimates for studies that experimentally examined the relationship between development rate and temperature for the stages from first instar to adult emergence. **Table S4.** Linear regression parameter estimates for studies that experimentally examined the relationship between the development rate and temperature for the life stages from hatch to pupation. Click here for file

Additional file 2: Figure S1 Meta-analysis of the effect of temperature, i.e. *B*_*1*_*-* the slope of the regression of temperature and development rate from hatch to emergence. (A) Forest plot of best model with random effect of publication author. (B) Funnel plot corresponding to plot (A). The weight of the study is indicated by the size of the square and the diamond indicates the overall effect estimate from the random effects model. Squares represent effect estimates of individual studies. Square size represents the weight given to the study in the meta-analysis, and the horizontal lines represent 95% confidence intervals. Estimated values and confidence intervals are written to the right of the plot. In the funnel plots, dots represent the residuals of the publication authors corresponding with the best model and their associated standard error. When the residuals fit within the light cone, it implies that heterogeneity in the main effect is successfully accounted for by the model. Click here for file

Additional file 3: Figure S2 Meta-analysis of the effect of temperature, i.e. *B*_*1*_*-* the slope of the regression of temperature and development rate from hatch to pupation. (A) Forest plot of best model with random effect of publication author. (B) Funnel plot corresponding to plot (A). See Figure S1 caption. Click here for file
