# Supplementary figures and images for: A meta-analysis of the factors influencing development rate variation in Aedes aegypti (Diptera: Culicidae)
Source: BMC Ecol. 2014 Feb 5;14:3. doi: 10.1186/1472-6785-14-3 (PMC3916798; doi:10.1186/1472-6785-14-3)

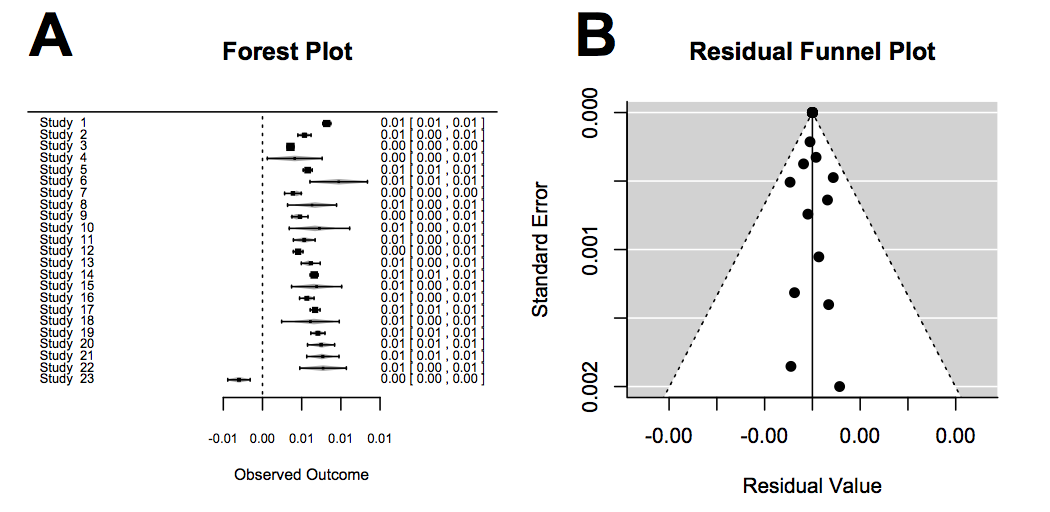

Supplement: Additional file 2: Figure S1 — Meta-analysis of the effect of temperature, i.e. B 1 - the slope of the regression of temperature and development rate from hatch to emergence. (A) Forest plot of best model with random effect of publication author. (B) Funnel plot corresponding to plot (A). The weight of the study is indicated by the size of the square and the diamond indicates the overall effect estimate from the random effects model. Squares represent effect estimates of individual studies. Square size represents the weight given to the study in the meta-analysis, and the horizontal lines represent 95% confidence intervals. Estimated values and confidence intervals are written to the right of the plot. In the funnel plots, dots represent the residuals of the publication authors corresponding with the best model and their associated standard error. When the residuals fit within the light cone, it implies that heterogeneity in the main effect is successfully accounted for by the model. [file 1472-6785-14-3-S2.png]

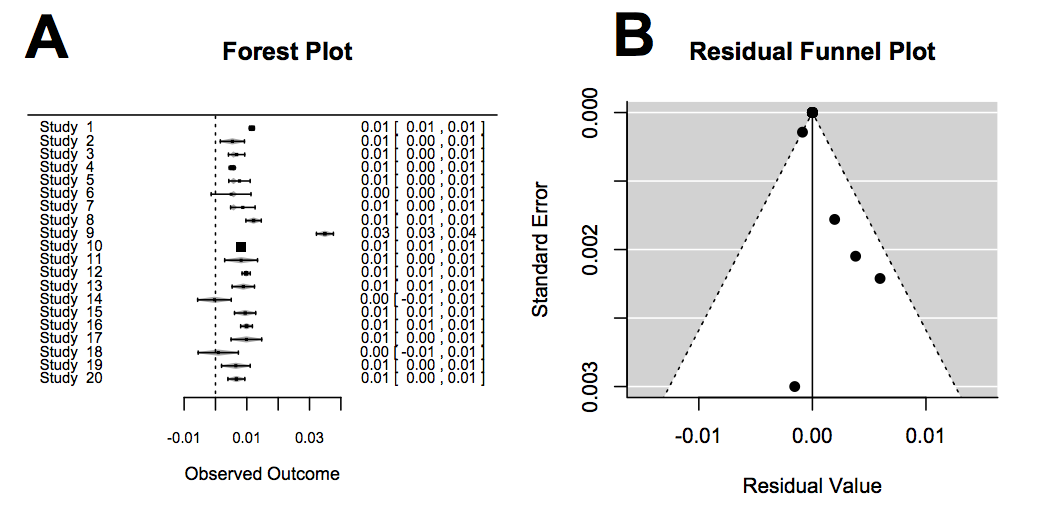

Supplement: Additional file 3: Figure S2 — Meta-analysis of the effect of temperature, i.e. B 1 - the slope of the regression of temperature and development rate from hatch to pupation. (A) Forest plot of best model with random effect of publication author. (B) Funnel plot corresponding to plot (A). See Figure S1 caption. [file 1472-6785-14-3-S3.png]
